# Loss of GFAT1 promotes epithelial-to-mesenchymal transition and predicts unfavorable prognosis in gastric cancer

**DOI:** 10.18632/oncotarget.9538

**Published:** 2016-05-21

**Authors:** Fangfang Duan, Dongwei Jia, Junjie Zhao, Weicheng Wu, Lingqiang Min, Shushu Song, Hao Wu, Lan Wang, Hongshan Wang, Yuanyuan Ruan, Jianxin Gu

**Affiliations:** ^1^ Key Laboratory of Glycoconjugate Research Ministry of Public Health, School of Basic Medical Sciences, Fudan University, Shanghai, P.R. China; ^2^ Department of Biochemistry and Molecular Biology, School of Basic Medical Sciences, Fudan University, Shanghai, P.R.China; ^3^ Institutes of Biomedical Sciences, Fudan University, Shanghai, P.R. China; ^4^ Department of General Surgery, Zhongshan Hospital, Fudan University, Shanghai, P.R. China

**Keywords:** GFAT1, gastric cancer, epithelial-to-mesenchymal transition, prognostic factor, TGF-β1

## Abstract

Gastric cancer remains the third leading cause of cancer-related mortality worldwide, and invasion and metastasis of gastric cancer represent the major reason for its poor prognosis. Glutamine: fructose-6-phosphate amidotransferase 1 (GFAT1) is the first and rate-limiting enzyme of hexosamine biosynthesis pathway (HBP). Nevertheless, the role of GFAT1 in gastric cancer is little investigated. In this study, we found that the expression of GFAT1 was decreased in gastric cancer. Low expression of GFAT1 was positively associated with vessel invasion, late T stage, lymph node metastasis, distant metastasis, advanced TNM stage and poor prognosis in patients with gastric cancer. Furthermore, *in vitro* and *in vivo* studies revealed that down-regulation of GFAT1 promoted epithelial-to-mesenchymal transition (EMT) and invasive activities in gastric cancer cells through inducing the expression of TGF-β1. The GFAT1 expression also significantly correlated with EMT-related factors in gastric cancer patients. Together, these findings indicate that GFAT1 functions as a novel suppressor of EMT and tumor metastasis in gastric cancer.

## INTRODUCTION

Gastric cancer remains the fifth most frequent and third leading cause of cancer-related mortality worldwide, though its incidence has decreased over the past six decades [1, 2]. The Asian countries account for the majority of gastric cancer cases, and almost 50% of the world's cases are diagnosed in China [3, 4]. Due to atypical symptoms in the early stages, most patients are diagnosed at advanced stage, when the 5-year survival rate ranges only from 4% to 20% for surgically resected cases [5]. The high rate of invasion and metastasis represents the major cause for its poor prognosis. It was reported that lymph node metastasis presented in more than 50% of gastric cancer patients when they were initially diagnosed [6], while peritoneum metastasis might be already present in 5% to 20% of patients undergoing gastric resection in curative intent [7].

Epithelial-mesenchymal transition (EMT) is a cellular mechanism known to occur during critical phases of embryonic development [8]. Similar, yet pathophysiological transitions occur during the progression of epithelial tumors, endowing cancer cells with increased motility and invasiveness to seed metastasis [9]. Multiple signaling pathways have been reported to orchestrate EMT process through modulating pleiotropically acting transcription factors (TFs), such as Snail, Twist and ZEB [10]. In response, cancer cells switch off the expression of epithelial markers, such as E-cadherin and catenins, and turn on mesenchymal markers, including Vimentin and fibronectin [10].

The hexosamine biosynthesis pathway (HBP) is a branch of the glucose metabolic pathway, consuming approximately 2–5% of the total glucose [11]. Flux through the HBP integrates carbohydrate, fat, protein and nucleotide metabolism, with the generation of uridine diphosphate N-acetylglucosamine (UDP-GlcNAc) which contributes to the aberrant glycosylation in different cancer types [12]. The limiting step of the HBP is catalyzed by glutamine: fructose-6-phosphate amidotransferase (GFAT) that converts fructose-6-phosphate to glucosamine-6- phosphate. There are two isoforms of GFAT encoded by separate genes, and GFAT1 is the major form ubiquitously expressed in different tissues and organs [13–15]. Though HBP has been considered to link glucose metabolism to malignant transformation [16], the expression and biological function of GFPT1 in gastric carcinoma remains little investigated. In this study, we found that GFAT1 was decreased in gastric cancer and suppressed EMT of tumor cells. Low expression of GFAT1 was identified as an independent factor that predicted unfavorable prognosis in gastric cancer patients.

## RESULTS

### The expression of GFAT1 is decreased in gastric cancer and associated with tumor progression

**Figure 1:**
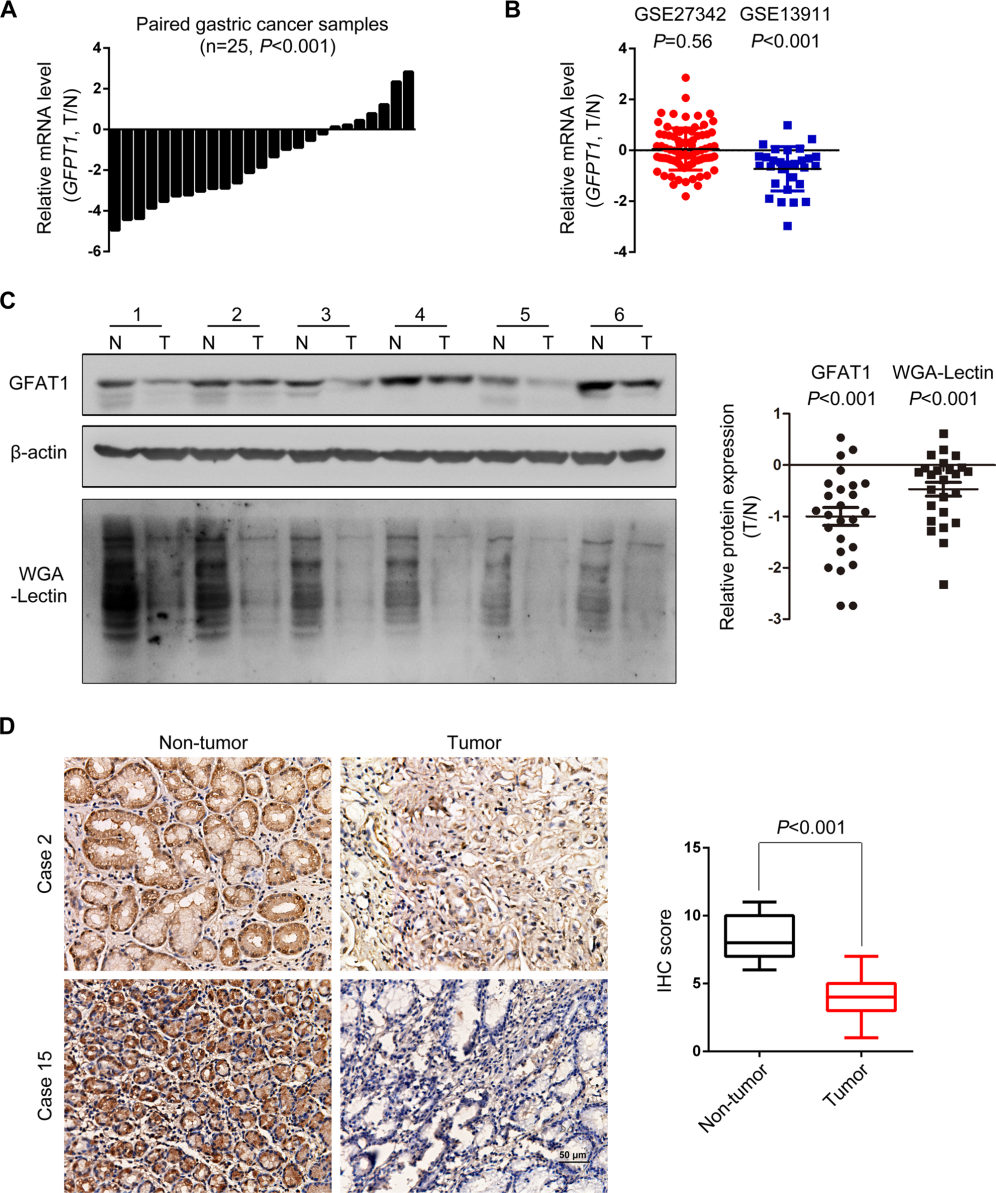
The expression of GFAT1 is decreased in gastric cancer (**A**) The mRNA expression of *GFPT1* in 25 pairs of gastric cancer tissues and adjacent normal mucosa was examined by real-time PCR analysis. Data shown are the log values of tumor vs normal. (**B**) Expression profiling of *GFPT1* mRNA in matched gastric cancer samples from GSE27342 and GSE13911 datasets. Data shown are the log values of tumor vs normal. (**C**) GFAT1 protein expression and WGA lectin staining in 25 paired gastric cancer samples was detected by western-blot. Blots shown are representative results in 6 cases. N, adjacent non-tumor sections; T, tumor sections. (D) GFAT1 protein expression in 15 paired gastric cancer samples was detected by IHC. Images shown are representative of GFAT1 staining in 2 paired gastric cancer tissues. The expression of GFAT1 in paired tumor tissues was compared by IHC scoring. In (B–C), data are represented as Means ± SD. In (**D**), data are represented as min-to-max bar graphs with median lines.

To understand whether GFAT1 was involved in gastric carcinogenesis, we first examined the mRNA expression of GFAT1 in paired fresh gastric cancer tissues. As shown in Figure 1A, the relative mRNA level of GFAT1 was dramatically decreased in gastric cancer tissues compared with paired normal gastric mucosa (*P* < 0.001). We also analyzed the GFAT1 mRNA expression in two reported datasets (GSE27342 and GSE13911) [17, 18]. Results showed that the mRNA expression of GFAT1 was remarkably decreased in gastric cancer samples from GSE13911 dataset, while no significant difference was observed in the GSE27342 dataset, suggesting the heterogeneity of gastric carcinoma (Figure 1B). Western blot analysis revealed that GFAT1 protein levels were remarkably down-regulated in tumor tissues by comparing with matched adjacent normal mucosa (Figure 1C). Decreased expression of GFAT1 was observed in 88% (22/25) cases (Figure 1C, right panel). Accordingly, wheat germ agglutinin (WGA) lectin blot also indicated that N-acetylglucosamine glycosylation was dramatically decreased in gastric cancer cases (Figure 1C). The GFAT1 expression and WGA lectin staining were also lower in gastric cancer cell lines, by comparing with those in normal gastric epithelial cell line GES-1 (Supplementary Figure 1). Moreover, immunohistochemistry (IHC) assay also indicated that the protein expression of GFAT1 was apparently lower in gastric cancer tissues than in non-tumor gastric mucosa (Figure 1D). We also examined the expression pattern of GFAT2, the other member of GFAT family, in gastric cancer cells and tissues. However, no protein expression of GFAT2 was detected (Supplementary Figure 2).

To explore whether GFAT1 was associated with tumor progression in clinical gastric cancer cases, a tissue microarray containing 211 gastric cancer samples was employed in immunohistochemistry assay to examine the relationship between GFAT1 expression and clinicopathological characteristics. The intratumoral GFAT1 expression in IHC was evaluated by CES scoring [19], and the high and low expression of GFAT1 was determined by ROC curve analysis. The association between GFAT1 expression and clinicopathological variables in gastric cancer patients was analyzed by chi-square test and listed in Table 1. Among the variables, low expression of GFAT1 was positively correlated with vessel invasion (*P* = 0.031), late T stage (*P* = 0.005), lymph node metastasis (*P* = 0.002), distant metastasis (*P* = 0.024) and advanced TNM stage (*P* < 0.001). These data suggest that low intratumoral GFAT1 expression is positively correlated with gastric cancer progression and metastasis.

**Table 1:** Correlation between GFAT1 expression and clinicopathological variables of 211 gastric cancer patients

Clinicopathological Variables	No.	GFAT1 expression		*p*-value^*^
		Low (*n* = 106) No. (%)	High (*n* = 105) No. (%)	
**Gender**				
Male Female	146 65	68 (46.6) 38 (58.5)	78 (53.4) 27 (41.5)	0.111
**Age (years)**				
< 60 ≥ 60	91 120	45 (49.5) 61 (50.8)	46 (50.5) 59 (49.2)	0.842
**Tumor site**				
Cardia Body Antrum	34 46 131	20 (58.8) 23 (50.0) 63 (48.1)	14 (41.2) 23 (50.0) 68 (51.9)	0.644
**Tumor diameter**				
< 4cm ≥ 4cm	101 110	48 (47.5) 58 (52.7)	53 (52.5) 52 (47.3)	0.450
**Lauren classification**				
Intestinal Diffuse Mixture	137 56 18	65 (47.4) 32 (57.1) 9 (50.0)	72 (52.6) 24 (42.9) 9 (50.0)	0.473
**Tumor differentiation**				
Well Moderately Poorly	4 32 175	2 (50.0) 14 (43.8) 90 (51.4)	2(50.0) 18(56.2) 85(48.6)	0.727
**Vessel invasion**				
Positive Negative	69 142	42 (60.9) 64 (45.1)	27(39.1) 78(54.9)	0.031
**T stage**				
T1 T2 T3 T4	20 24 74 93	9 (45.0) 6 (25.0) 33 (44.6) 58 (62.4)	11 (55.0) 18 (75.0) 41 (55.4) 35 (37.6)	0.005
**N stage**				
N0 N1 N2 N3	54 32 40 85	18 (33.3) 13 (40.6) 20 (50.0) 55 (64.7)	36 (66.7) 19 (59.4) 20 (50.0) 30 (35.3)	0.002
**Distant metastasis**				
Yes No	5 206	5 (100) 101 (49.0)	0 (0) 105 (51.0)	0.024
**TNM stage**				
I II III IV	26 55 125 5	7 (26.9) 19 (34.5) 75 (60.0) 5 (100)	19 (73.1) 36 (65.5) 50 (40.0) 0 (0)	< 0.001

* Pearson chi-square tests.

*P* < 0.05 indicates the differences have statistical significance.

### Correlations between GFAT1 expression and prognosis in gastric cancer patients

**Figure 2:**
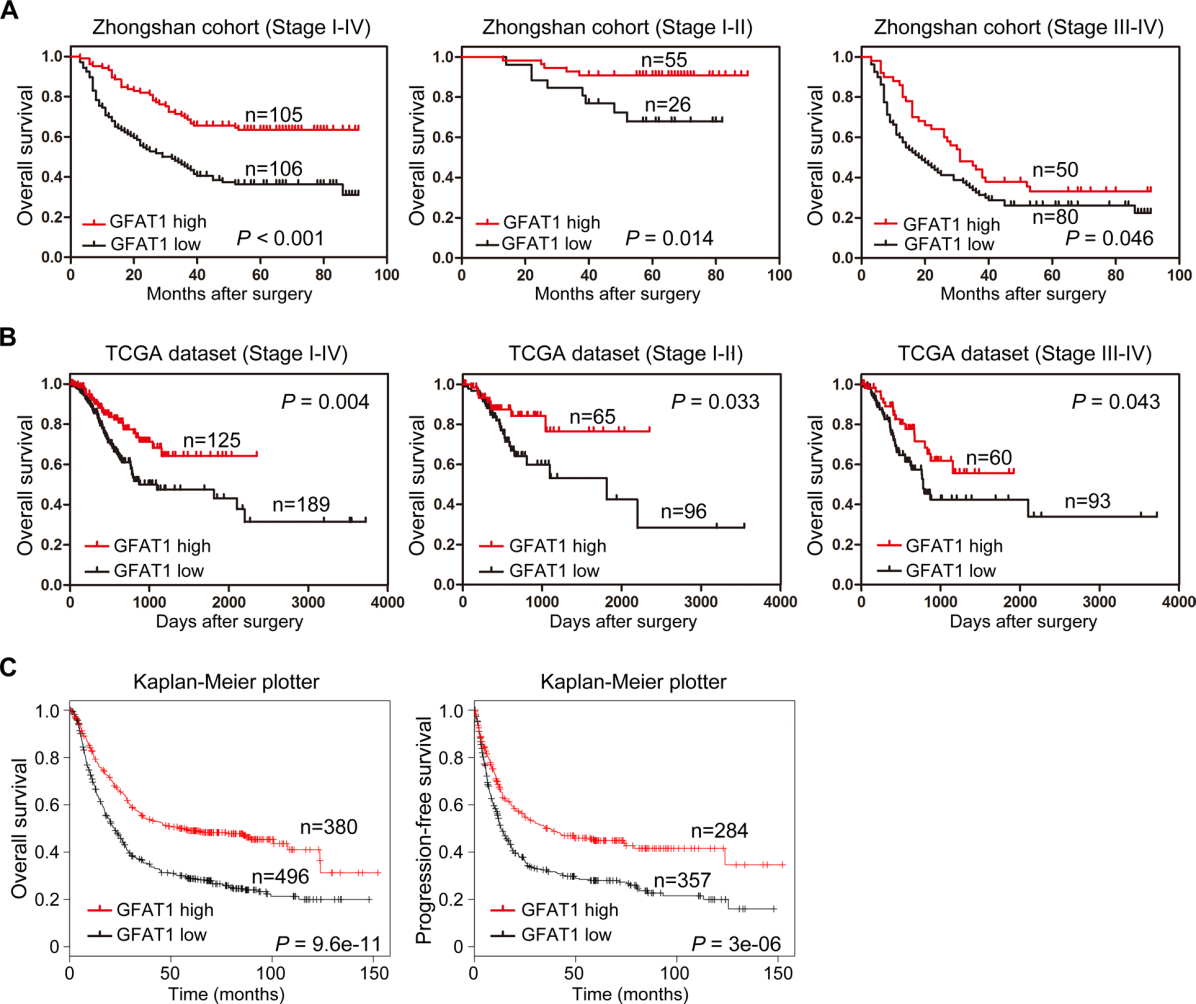
Kaplan-Meier survival analysis for overall survival of gastric cancer patients according to the GFAT1 expression (**A**–**B**) The association of GFAT1 expression with overall survival was examined by Kaplan-Meier analysis in gastric cancer patients from Zhongshan cohort (A) and TCGA dataset (B), respectively. Total patients in each cohort were also divided into TNM I-II stage and TNM III-IV stage subgroups for analysis. (**C**) The association of GFAT1 expression with overall survival and progression-free survival was examined by Kaplan-Meier analysis in gastric cancer patients using the online survival analysis software (http://www.kmplot.com/analysis/index.php?p=service&cancer=gastric).

We next explored the relationship between GFAT1 expression and overall survival by utilizing Kaplan-Meier analysis and Log-rank test. Results demonstrated that low expression of GFAT1 in tumor tissues showed a survival disadvantage for gastric cancer patients in both our cohort and TCGA dataset (Figure 2A and 2B). To further evaluate the efficiency of GFAT1 expression on stratifying patients with different TNM stages, we divided the patients into early (I-II) and advanced (III-IV) groups, respectively. In our cohort, the GFAT1 expression showed statistically significant value in predicting the outcome of gastric cancer patients in both TNM I+II and TNM III+IV subgroups (Figure 2A). Similar predictive value of GFPT1 for overall survival in both subgroups was also observed in the gastric cancer patients from the TCGA dataset (Figure 2B).

We also explored the correlation between GFAT1 expression and the survival of gastric cancer patients by using an online survival analysis software (http://www.kmplot.com/analysis/index.php?p=service&cancer=gastric), which integrated reported microarray datasets [20]. Results also demonstrated that low expression of GFAT1 was significantly associated with shorter overall survival as well as shorter progression-free survival in gastric cancer patients (Figure 2C). These data suggest that low GFAT1 expression correlates with poor prognosis for patients with gastric cancer.

To identify the prognostic significance of clinicopathological factors for overall survival, univariate Cox analysis was conducted. T stage (*P* < 0.001), N stage (*P* < 0.001), distant metastasis (*P* = 0.001), TNM stage (*P* < 0.001), vessel invasion (*P* = 0.001), and GFAT1 expression (*P* < 0.001) were identified as risk factors that were correlated with the overall survival of gastric cancer patients (Table 2). Further adjustment of covariate factors by using multivariate Cox analysis identified T stage (*P* = 0.003), TNM stage (*P* < 0.001) and GFAT1 expression (*P* = 0.019) as independent prognostic factors for gastric cancer patients (Table 2).

**Table 2:** Univariate and multivariate Cox regression analysis of clinicopathological characteristics influencing the overall survival of gastric cancer patients

Variables	Univariate	Multivariate	
	*P*-value	HR (95% CI)	*P*-value
**Gender**			
Female vs Male	0.619		
**Age**			
< 60 vs ≥ 60	0.621		
**Tumor site**			
Cardia + Body vs Antrum	0.239		
**Tumor diameter**			
< 4 cm vs ≥ 4 cm	0.216		
Lauren classification			
Intestinal + Mixture vs Diffuse	0.747		
**Tumor differentiation**			
Well + Moderately vs Poorly	0.574		
**T stage**			
T1–T2 vs T3–T4	< 0.001	6.110 (1.882–19.839)	0.003
**N stage**			
N0 vs N1–N3	< 0.001		0.094
**Distant metastasis**			
Yes vs No	0.001		0.346
**TNM stage**			
I–II vs III–IV	< 0.001	3.320 (2.134–5.167)	< 0.001
**Vessel invasion**			
Positive vs Negative	0.001		0.281
**GFAT1 expression**			
High vs Low	< 0.001	0.614 (0.409–0.922)	0.019

CI, Confidence interval; HR, Hazard ratio.

### GFAT1 suppresses epithelial-to-mesenchymal transition in gastric cancer cells

**Figure 3:**
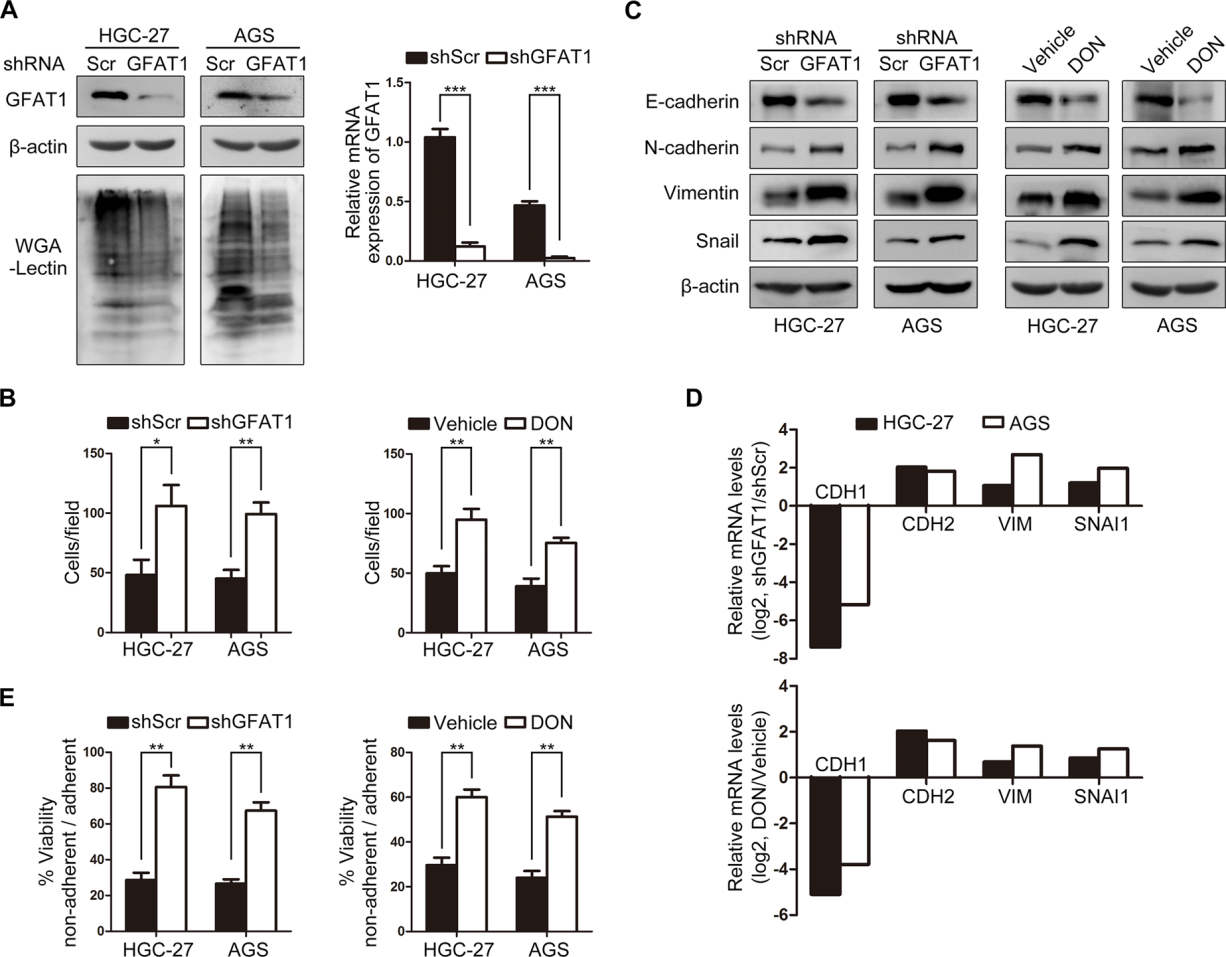
GFAT1 suppresses epithelial-to-mesenchymal transition and invasive activities in gastric cancer cells (**A**) The GFAT1 expression pattern and WGA lectin staining in gastric cancer cells stably transfected with scramble (Scr) or GFAT1 shRNA. (**B**) The effect of GFAT1 specific shRNA or GFAT1 inhibitor DON on the invasion of gastric cancer HGC-27 and AGS cells by transwell analysis. (**C**–**D**) The effect of GFAT1 specific shRNA or GFAT1 inhibitor DON on the expression of EMT markers in gastric cancer HGC-27 and AGS cells was evaluated by western-blot (C) and real-time PCR (D) analysis, respectively. (**E**) The effect of GFAT1 specific shRNA or GFAT1 inhibitor DON on the relative viability of gastric cancer HGC-27 and AGS cells by anoikis assay. *P < 0.05; **P < 0.01; ***P < 0.001. In (A–B) and (E), data are represented as Means ± SD.

To gain a mechanistic understanding of the potential role of GFPT1 in modulating gastric cancer metastasis, gastric cancer cells stably expressing GFAT1 shRNA were generated (Figure 3A). We found that knock-down of GFAT1 induced the invasive potential of tumor cells (Figure 3B, left panel). In addition, administration of GFAT1 inhibitor 6-diazo-5-oxo-l-norleucine (DON) also induced more invasive gastric cancer cells (Figure 3B, right panel).

Epithelial-to-mesenchymal transition (EMT) enables cancer cells with invasive and metastatic properties in tumor progression [21]. We next examined the potential effect of GFAT1 on the expression of EMT markers. Western-blot analysis revealed that in GFAT1-depleted HGC-27 and AGS cells, the protein level of epithelial marker E-cadherin was repressed, while the expression of mesenchymal marker N-cadherin and Vimentin as well as transcriptional repressor Snail were markedly increased (Figure 3C). Real-time PCR analysis showed that knock-down of GFAT1 blocked mRNA level of E-cadherin, and up-regulated N-cadherin, Vimentin as well as Snail mRNA expression (Figure 3D). Similar effects on the expression of EMT markers were also observed in DON-treated cells (Figure 3C and 3D). In addition, inhibition of GFAT1 with specific shRNA or DON also promoted anoikis resistance in gastric cancer cells by using ultra-low attachment plate (Figure 3E). Together, these results suggest that GFPT1 functions as a suppressor of epithelial-to-mesenchymal transition in gastric cancer cells.

### Loss of GFAT1 promotes epithelial-to-mesenchymal transition through inducing TGF-β1 expression

**Figure 4:**
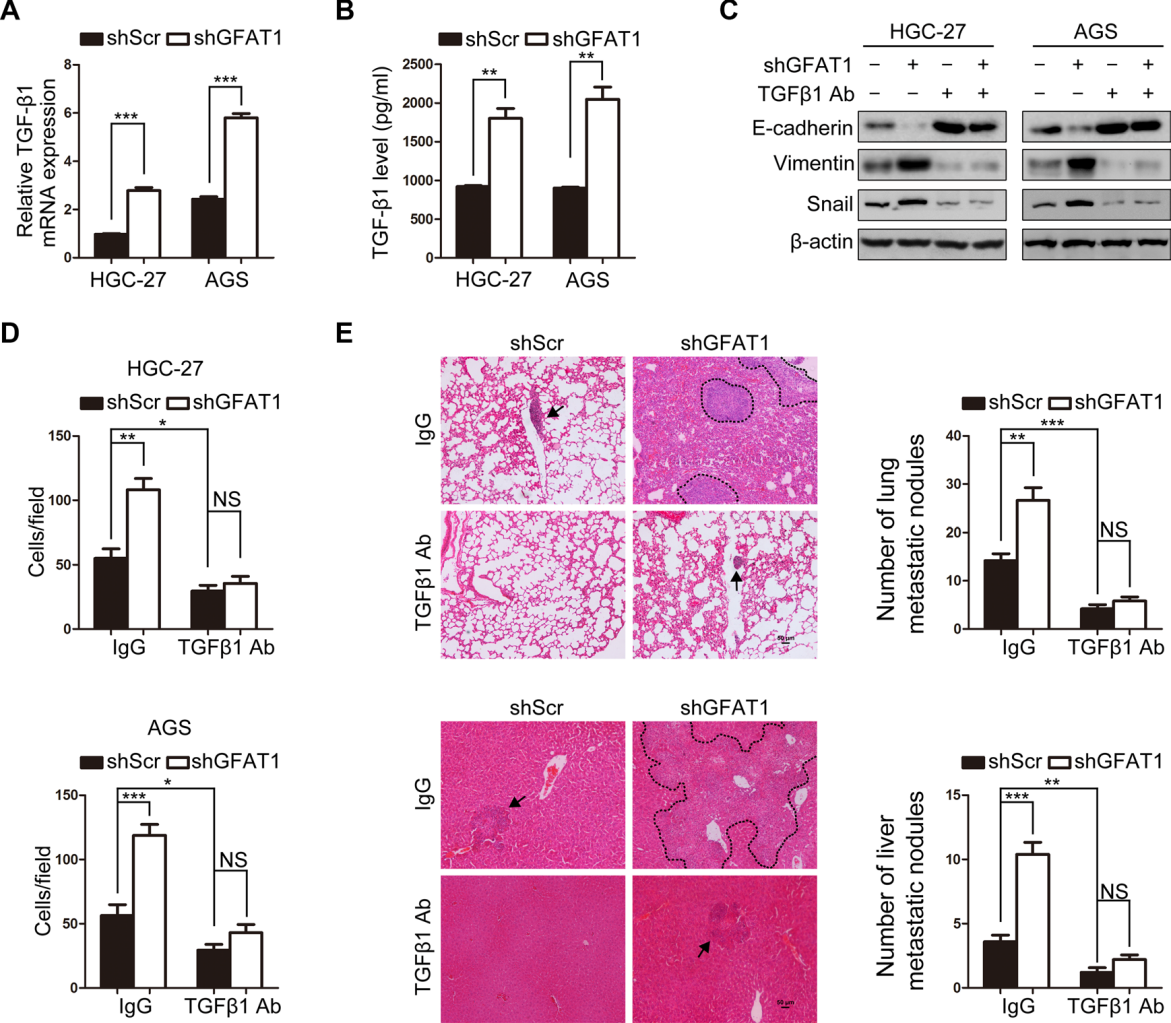
Loss of GFAT1 promotes epithelial-to-mesenchymal transition through inducing TGF-β1 expression in gastric cancer (**A**) The effect of GFAT1 specific shRNA on the mRNA expression of TGF-β1 in gastric cancer HGC-27 and AGS cells was detected by real-time PCR analysis. (**B**) The effect of GFAT1 specific shRNA on the levels of TGF-β1 in the culture supernatant of gastric cancer HGC-27 and AGS cells was detected by ELISA. (**C**) The effect of TGF-β1 neutralizing antibody on the expression of EMT markers in control or GFAT1-depleted gastric cancer cells was examined by western blot. (**D**) The effects of TGF-β1 neutralizing antibody on the invasion of control or GFAT1-depleted HGC-27 and AGS cells were examined by transwell analysis. (**E**) Stable AGS cells were injected into the lateral tail vein of mice. After the sacrifice of mice, lung and liver tissues was collected. Images were representatives of HE staining in lung (upper panel) and liver (lower panel) tissue sections from each group (scale bar, 50 μm). Micrometastatic lesions were indicated with arrows or dotted line. Numbers of lung and liver metastatic foci in each group were also counted. *P < 0.05; **P < 0.01; ***P < 0.001; NS, no significance. In (A–B) and (D–E), data are represented as Means ± SD.

It has been well characterized that transforming growth factor-beta 1 (TGF-β1) is a potent inducer of EMT during cancer pathogenesis [8]. And we found that knock-down of GFAT1 with specific shRNA increased the mRNA expression of TGF-β1 in both HGC-27 and AGS cells (Figure 4A). ELISA assay also revealed that the levels of secreted TGF-β1 were up-regulated in the supernatant of gastric cancer cells with GFAT1 depletion (Figure 4B). We next assessed whether loss of GFAT1 stimulated the EMT of gastric cancer cells through TGF-β1. As shown in Figure 4C, administration of TGF-β1 neutralizing antibody blocked the increased levels of Snail and Vimentin as well as the down-regulation of E-cadherin in GFAT1-depleted tumor cells. In addition, TGF-β1 inhibition also dramatically repressed GFAT1 shRNA-induced tumor invasion *in vitro* (Figure 4D).

We also injected stable AGS cells into lateral tail vein of nude mice and examined tumor metastasis *in vivo*. After the sacrifice of mice, we found that more and larger micrometastatic lesions were microscopically detected in the lungs and livers of nude mice inoculated with GFAT1 shRNA-transfected AGS cells (Figure 4E). Meanwhile, inhibition of TGF-β1 using its specific neutralizing antibody remarkably abrogated the formation of micro-tumor lesions and metastatic nodules in lungs and livers from mice bearing GFAT1-depleted AGS cells (Figure 4E). These results suggest that loss of GFAT1 promotes epithelial-to-mesenchymal transition and invasive activities in gastric cancer through inducing TGF-β1 expression.

### Correlated expression of GFAT1 with EMT-related factors in gastric cancer

**Figure 5:**
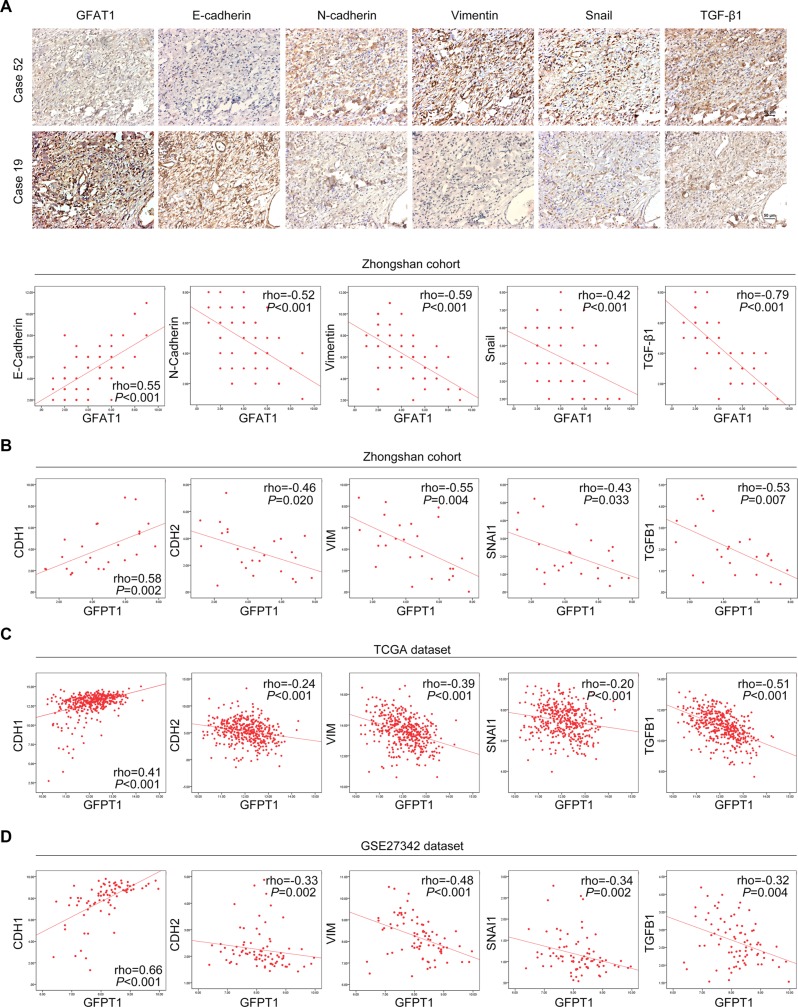
Correlated expression of GFAT1 with EMT-related factors in gastric cancer (**A**) The expression of EMT-related factors E-cadherin, N-cadherin, Vimentin, Snail and TGF-β1 in gastric cancer tissues from Zhongshan cohort was examined by IHC, and the correlation between their expression and GFAT1 was assessed individually. Representative images of IHC staining were shown in the upper panel. (**B**–**D**) The correlated expression of *GFPT1* mRNA level with transcript levels of *CDH1*, *CDH*2, *VIM*, *SNAI1* and *TGFB1* in Zhongshan cohort (B) as well as in TCGA (C) and GSE27342 (D) datasets.

Since loss of GFAT1 contributed to the epithelial-to-mesenchymal transition in gastric cancer cells, we next determined whether expression of GFAT1 was correlated with EMT-related factors in gastric cancer tissues. As shown in Figure 5A, immunohistochemistry assay revealed that the protein expression of GFAT1 was positively correlated with E-cadherin staining and negatively associated with the levels of N-cadherin, Vimentin, Snail as well as TGF-β1 in our cohort of gastric cancer samples. Similar correlation of GFAT1 mRNA expression with the transcript levels of EMT-related factors were also observed in our cohort as well as in TCGA and GSE27342 datasets (Figure 5B–5D). These results suggest that the expression of GFAT1 is negatively associated with epithelial-to-mesenchymal transition in gastric cancer patient samples.

## DISCUSSION

Increased glucose uptake and metabolism have been well recognized as a common feature of cancer cells. Although most cellular glucose is metabolized by glycolysis, 2–5% is channeled into the HBP and isomerized in two enzymatic steps to yield fructose-6-phosphate [11]. GFAT then acts to convert fructose-6-phosphate to glucosamine-6-phosphate in the HBP's rate-limiting step. Though the precise function of GFAT in tumor progression remains not defined, several previous studies have shown the anti-neoplastic properties of GFAT inhibitors DON and azaserine [22, 23], suggesting a potential role of GFAT in driving tumorigenesis. However, in our study, we found that GFAT1 functioned as a suppressor of EMT in transformed gastric mucosa, and low expression of GFAT1 predicted unfavorable prognosis in gastric cancer patients.

The regulation of GFAT1 expression has been explored previously. It has been reported that GFAT1 gene undergoes a late transcriptional response to epidermal growth factor, and this effect is repressed by high glucose concentration [24]. GFAT1 could also be controlled at post-translational level since it has a relatively short half-life of 1 h [25]. The activity of GFAT1 is tightly regulated. It is reported that cAMP-dependent protein kinase (PKA) phosphorylates GFAT1 at serine 235 and thereby inhibits its activity [26]. There is also allosteric feedback inhibition of GFAT1 activity by its product glucosamine 6-phosphate and HBP end product, UDP-GlcNAc [27]. These studies may provide basis for better understanding the regulation of GFAT1 expression and activity in gastric cancer in future research.

In this study, we identified TGF-β1 as a key factor involved in GFAT1 silencing-induced EMT in gastric cancer cells. Members of the TGF-β family are the main and the best-characterized inducers of EMT during the course of embryonic development and tumor pathogenesis [8]. Interestingly, it has been reported that GFAT1 promotes TGF-β1 expression in mesangial cells and fibroblasts [28, 29], and PKC/MAPK p38 cascade is involved in GFAT-1-inducd TGF-β1 up-regulation [30]. However, we found that GFAT1 suppressed TGF-β1 expression in gastric cancer cells, and the negative correlation between GFAT1 and TGF-β1 levels was also confirmed in patient samples. It has been well recognized that GFAT1 controls the generation of UDP-GlcNAc as the rate-limiting enzyme of HBP. UDP-GlcNAc is then utilized in N- and O-linked glycosylation that modifies various kinds of proteins, including transmembrane receptors, signaling molecules and transcription factors. Therefore, the different glycosylation spectrum may determine the regulatory effect of GFAT1 on TGF-β1 expression in various types of cells.

Dysregulation of GFAT1 has been reported in several kinds of diseases, including diabetes and cancer. A previous study indicates that GFAT activity is increased in the NIDDM (non-insulin dependent diabetes mellitus) patients and contributes to glucose toxicity and insulin resistance [31]. GFAT may be causally involved in the development of diabetic vascular disease, particularly diabetic nephropathy [32]. Recent research have also shown that GFAT1 expression is correlated with the tumor progression. Study of triple-negative breast cancer shows that GFAT1 is a prognostic marker that predicts worse progression-free survival and overall survival in patients [33]. In ER-positive breast cancers, the protein levels of GFAT1 in relapse breast cancer patients are also increased compared to non-relapse patients [34]. Moreover, polymorphisms of *GFPT1* is significantly associated with the risk and overall survival in pancreatic cancer [35, 36]. Future studies may focus on the molecular mechanisms how GFAT1 is involved in the development of these kinds of diseases.

To date, gastric cancer remains the third leading cause of cancer-related mortality worldwide [1, 2]. The onset and progression of gastric cancer are precipitated by various genetic alterations. Therefore, molecular approaches are urgently needed in understanding tumor progression, in discovering novel biomarkers, and in determining effective therapies for use in clinical settings. In this study, our data indicate that GFAT1 functions as a suppressor of EMT in gastric cancer, and suggest GFAT1 as a new biomarker to establish the risk and prognosis of gastric cancer and to help in the selection of therapeutic modalities in clinical practice.

## MATERIALS AND METHODS

### Patient samples

The use of human tissue samples and clinical data was approved by the ethics committee of Fudan University. All donors were informed of the aim of the study and gave consent to donate their samples. For tissue microarray detection, tumor specimens were obtained from 211 gastric cancer patients who underwent surgical resection without preoperative treatment from 2004 to 2008, at Department of General Surgery, Zhongshan Hospital, Fudan University, Shanghai, China. The diagnosis of gastric carcinoma was confirmed by pathologic examination. Staging data were according to the 7th edition of the AJCC Cancer Staging Manual [5]. All the patients' demographic characteristics, date of surgery, tumor stage, surgical and medical treatment methods, survival time, and other relevant data were extracted from hospital records. The independent group of 25 gastric cancer samples was also collected at Department of General Surgery, Zhongshan Hospital, Fudan University, Shanghai, China.

### Cells and reagents

All cell lines were purchased from Cell Bank of Type Culture Collection of Chinese Academy of Sciences, Shanghai Institute of Cell Biology, Chinese Academy of Sciences. Cells were cultured in RPMI 1640 or D-MEM supplemented with 10% fetal bovine serum (FBS) (Cat# 16000-044, Gibco) at 37°C in a humidified atmosphere containing 5% CO_2_.

GFAT1 antibody (Cat# ab176775) was purchased from Abcam (Cambridge, MA, USA). Vimentin (Cat# 5741), Snail (Cat# 3879), and β-actin (Cat# 3700) antibodies were purchased from Cell Signaling Technology (Beverly, MA, USA). E-cadherin (Cat# sc-71008 and sc-8426) and N-cadherin (Cat# sc-8424) antibodies were purchased from Santa Cruz Biotechnology (Dallas, TX, USA). 6-Diazo-5-Oxo-L-Norleucine (DON, Cat# D2141) and WGA (Wheat germ agglutinin, Cat# L3892) lectin were purchased from Sigma-Aldrich (St Louis, MO, USA). Snail antibody (Cat# 13099-1-AP) was purchased from ProteinTech (Chicago, IL, USA). GFAT2 antibody (Cat# 40023) was purchased from SAB (Pearland, TX, USA). TGF-β1 neutralizing antibody (Cat# MAB1835) was purchased from R&D Systems (Minneapolis, MN, USA). AlamarBlue^®^ cell viability reagent (Cat# DAL1025) was purchased from Thermo Fisher (Waltham, MA, USA). Ultra-low attachment 96-well plates were purchased from Corning (Corning, NY, USA).

### Tissue microarray (TMA)

A series of TMA containing gastric cancer samples were constructed as described in our previous report [19]. Briefly, all the gastric cancer tissues were reviewed by pathologist, and representative areas free from necrotic and hemorrhagic materials were pre-marked in the paraffin blocks. For each sample, 2-mm core was punched from the donor blocks, and transferred to the recipient paraffin block at defined array positions using a tissue microarray instrument (Manuel Tissue Arrayer, Beecher Instruments, Maryland, USA). Several serial sections (4 μm in thickness) were cut from all TMA, one of which was stained with hematoxylin-eosin as reference.

### Immunohistochemical staining and scoring

Immunohistochemistry was performed using a two-step procedure following the protocol recommended by Dako REAL^™^ EnVision^™^ Detection System, Peroxidase/DAB+ (Cat# K5007, Dako, Denmark). To ensure antibody specificity, control slides were incubated either in the absence of primary antibody or with a nonspecific IgG antibody. Positive brownish cytoplasm immunostaining in more than 5% of tumor cells was the criterion for immunostaining positivity for GFAT1. Slides were assessed by an experienced and independent pathologist blinded to the patient's status. To obtain an IHC score that takes into account the IHC signal intensity and the frequency of positive cells, a composite expression scores (CES) with full range from 0 to 12 was generated as described in our previous report [19].

### Short hairpin RNA

Transduction of short hairpin RNA (shRNA) targeting human GFAT1 (Cat# sc-60681-SH, Santa Cruz, Dallas, TX, USA) into gastric cancer cells was carried out by using X-tremeGENE HP DNA Transfection Reagent (Cat# 06366236001, Roche Applied Science, Penzberg, Germany), according to the manufacturer's instructions. Puromycin dihydrochloride was used for the selection of stable clones.

### *In vitro* invasion assays

Transwell invasion assays were performed in 24-well transwell plates (8 μm pore size) according to the manufacturer's instructions (Cat# 3422, Corning, New York, NY, USA). The upper chamber was filled with 5 × 10^4^ cells in basic culture medium without serum. The lower chamber was coated with BD Matrigel Basement Membrane Matrix (Cat# 354234, BD Biosciences, CA, USA), and filled with culture medium containing 20% FBS. After incubation for 30 h at 37°C, non-invading cells on the upper side of the chamber were removed, and invading cells on the lower surface of the membrane were fixed with 4% paraformaldehyde and stained with 0.1% crystal violet. The number of invading cells was counted in four randomly selected microscopic fields.

### *In vitro* anoikis studies

Anoikis studies were performed as described previously [37]. Briefly, cells were plated (3000 cells/well) onto 96-well tissue culture plates (adherent culture) or ultra-low attachment plates (non-adherent culture) for 3 days. Cell viability was quantified with alamar blue on a BioTek plate reader. Anoikis resistance was indicated by the ratio of signal in nonadherent culture versus adherent culture.

### Animal studies

Animal experiments were performed as described in our previous report [38]. Briefly, 4-week-old male BALB/C nude mice were obtained from Shanghai Laboratory Animal Center of Chinese Academy Sciences and housed in a specific pathogen-free room. Mice were primed with an injection of 2 × 10^6^ stable AGS cells in 150 μl of PBS into the lateral tail vein of mice. IgG (4 mg/kg) or TGF-β1 neutralizing antibody (4 mg/kg) were given i.p. three times per week. At day 56, all mice were sacrificed. The lung and liver tissues were fixed and embedded with paraffin, followed by HE staining. Animal care and experiments were performed in strict accordance with the “Guide for the Care and Use of Laboratory Animals” prepared by the National Academy of Sciences and published by the National Institutes of Health, and were approved by the ethics committee of Fudan University.

### Real-time polymerase chain reaction (PCR)

Cells were harvested, and total RNA was extracted using TRIzol Reagent (Cat# 15596-026, Gibco BRL and Life Technologies). RNA was reversely transcribed to cDNA by PrimeScript RT reagent Kit (Cat# DRR037A, Takara, Tokyo, Japan) in accordance with the manufacturer's instructions. Real-time PCR was performed by ABI StepOne Plus (Applied Biosystems) with the use of SYBR Premix Ex Taq (Cat# DRR041A, Takara, Tokyo, Japan). The primers were obtained from Origene (Rockville, MD, USA).

### Statistical analysis

Results are presented as Means ± SD. CES analysis was performed with nonparametric methods. The optimal cut-off value of CES is determined by ROC curve analysis. Categorical data were analyzed using the χ^2^ test. Differences between groups were determined using two-tailed student's *t* test. Correlation of GFPT1 expression with EMT-related factors was analyzed using nonparametric Spearman's *ρ* test. The Kaplan-Meier method was used to determine survival probability and differences were assessed by the log-rank test. Statistical significance was set at two-sided *P* < 0.05. All analysis was performed using SPSS 13.0 software (Chicago, IL, USA).

## SUPPLEMENTARY MATERIALS FIGURES


