# Supplementary material for: Loss of GFAT1 promotes epithelial-to-mesenchymal transition and predicts unfavorable prognosis in gastric cancer
Source: Oncotarget. 2016 May 21;7(25):38427–39. doi: 10.18632/oncotarget.9538 (PMC5122401; doi:10.18632/oncotarget.9538)
Supplement: Supplementary file 1 [file oncotarget-07-38427-s001.pdf]

Loss of GFAT1 promotes epithelial-to-mesenchymal transition and predicts unfavorable prognosis in gastric cancer

Supplementary Materials

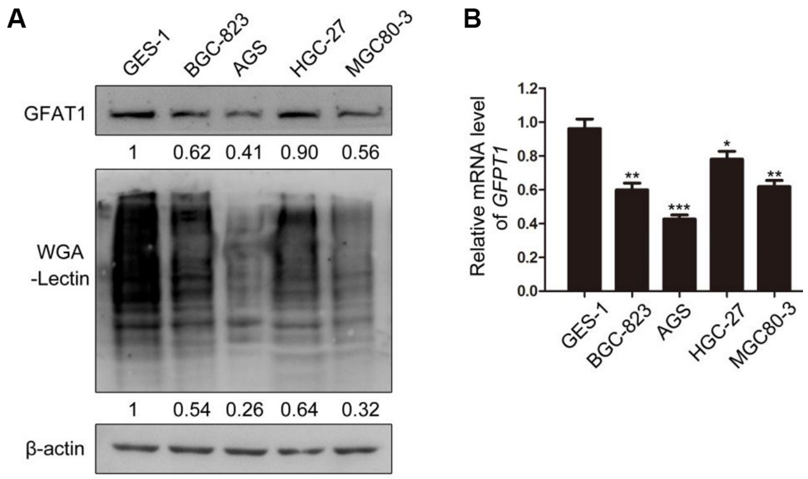

Supplementary Figure S1: The expression of GFAT1 was decreased in gastric cancer cell lines. (**A**) The GFAT1 protein expression and WGA lectin staining in normal gastric epithelial cell line GES-1 and four kinds of gastric cancer cell lines (BGC-823, AGS, HGC-27 and MGC80-3). Quantifications were made by comparing with β-actin. (**B**) The GFPT1 mRNA levels in GES-1 cells and four kinds of gastric cancer cell lines by real-time PCR assay. **P* < 0.05; ***P* < 0.01; ****P* < 0.001.

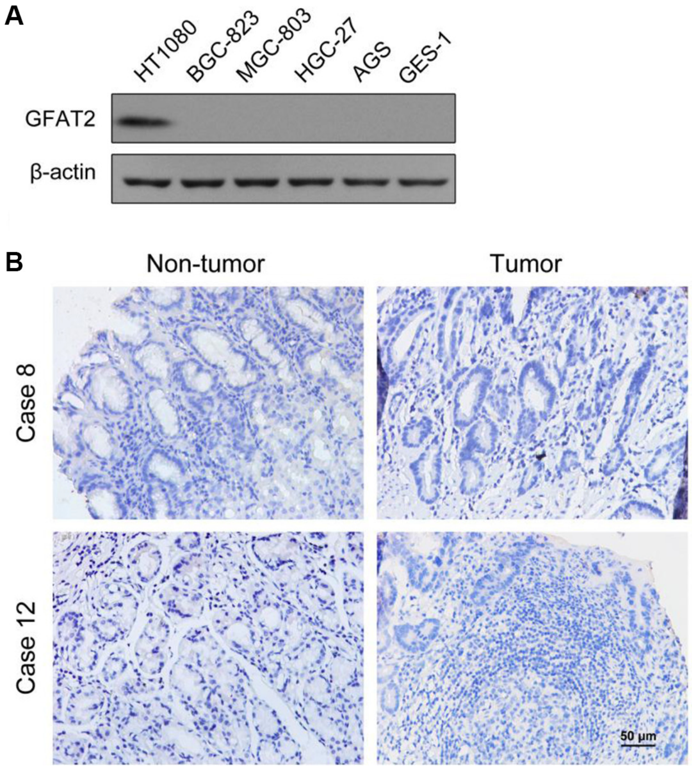

Supplementary Figure S2: The expression pattern of GFAT2 in gastric cancer. (**A**) The GFAT2 protein expression in normal gastric epithelial cell line GES-1 and four kinds of gastric cancer cell lines (BGC-823, AGS, HGC-27 and MGC80-3). HT1080 cells were used as positive control. (**B**) The GFAT2 protein expression in 15 paired gastric cancer samples by IHC analysis. Representative images from 2 cases were shown. Scale bar, 50 μm.
